# A fully transanal endoscopic approach for large post‐anastomotic high rectovaginal fistulas: An IDEAL stage 1 technical note

**DOI:** 10.1111/codi.70419

**Published:** 2026-02-26

**Authors:** Alfonso Lapergola, Federica Alicata, Paul Hag, Fatema Alhammadi, Pietro Riva, Elisa Reitano, Antonio D'Urso, Didier Mutter

**Affiliations:** ^1^ Visceral and Digestive Surgery Department Nouvel Hôpital Civil, University Hospital of Strasbourg Strasbourg France; ^2^ IRCAD (Research Institute against Digestive Cancer) Strasbourg France; ^3^ IHU (Institut Hospitalo‐Universitaire/University Hospital Institute) Strasbourg France; ^4^ Deparment of Surgery University of Rome “Tor Vergata” Rome Italy; ^5^ Department of Surgery Sapienza University Rome Italy

**Keywords:** high rectovaginal fistula, post‐anastomotic fistula, rectovaginal fistula, TEO platform, transanal endoscopic surgery

## Abstract

**Background:**

High rectovaginal fistulas (RVFs) remain a challenging condition, often requiring transabdominal surgery associated with significant morbidity, particularly in patients with hostile abdomens and multiple prior pelvic operations. Minimally invasive, video‐assisted and sphincter‐preserving alternatives have been described for mid and low RVF but exceptionally reported for high and large defects.

**Technique:**

We describe a fully transanal endoscopic approach for the repair of large, high post‐anastomotic rectovaginal fistulas using the transanal endoscopic operation (TEO®) platform. According to the IDEAL framework, this represents an IDEAL stage 1 procedure aimed at demonstrating technical feasibility and immediate safety. The technique involves six standardized steps, including marking of the defect, precise endoscopic dissection to achieve adequate reciprocal mobilization of the rectal and vaginal walls, followed by tension‐free, layered closure of both defects under stable endoscopic vision. A concise instructional video accompanies the manuscript to illustrate the key technical steps.

**Results:**

The technique was applied in a representative patient with a large high post‐anastomotic RVF and a hostile surgical history. The procedure was completed successfully without intraoperative complications. Postoperative imaging and endoscopic evaluation confirmed complete closure of the fistula. The postoperative course was uneventful, and no recurrence was observed after 2 years of follow‐up.

**Conclusion:**

This technical note demonstrates the feasibility of a fully transanal endoscopic approach for selected large and high rectovaginal fistulas. By avoiding transabdominal access, this organ‐preserving technique may represent a valuable option in experienced centres for patients in whom conventional approaches are associated with high morbidity. Further evaluation in larger series is warranted.

## BACKGROUND

Rectovaginal fistulas (RVFs) are aberrant epithelialized connections between the rectum and vagina, arising from postoperative complications, inflammatory bowel disease, obstetrical trauma or pelvic irradiation. Patients may experience vaginitis, passage of flatus or faeces through the vagina and severe skin excoriation, with significant interference in daily activities and sexual life, sometimes leading to psychological consequences.

Given their heterogeneity, a customized treatment plan is essential (as ‘one‐technique‐fits‐all’ strategies are seldom effective), considering fistula location (high or mid/low location, size, sphincter involvement), aetiology (post‐surgical, gynaecological, IBD‐related, radiation‐induced, post‐traumatic), prior surgical history, surgeon experience and available equipment.

Surgical options include transvaginal, transrectal, transperineal or transabdominal approaches depending on complexity, fistula height and/or the presence of accompanied sphincter injury.

Complexity is typically categorized as ‘complex or simple’ and height as ‘mid/low or high’, though no universally accepted classification exists. Generally, the diagnosis and complexity are confirmed and classified through endoscopic examination and dedicated imaging, such as pelvic MRI.

While low RVFs are usually managed transanally, transvaginally or transperineally, high RVFs often require transabdominal access, sometimes necessitating major rectal resection and ‘pull‐through’ coloanal anastomosis when defects exceed 2 cm [1].

Recent literature highlights the potential of transanal endoscopic repair as a minimally invasive alternative, though mostly in low–mid RVFs [2, 3, 4, 5, 6, 7, 8, 9, 10, 11, 12, 13] (Table 1).

**TABLE 1:** Details and results of previous transanal endoscopic surgical repair of RVFs reports.

	No. of patients	Aetiology	Location	Diverting stoma	Endoscopic platform	Follow‐up	Results
Darwood et al. [3], 2008	1	Surgery with radiation	Mid‐low	Yes, loop ileostomy	TEM	6 months	Successful closure
Vavra et al. [2], 2009	1	Trauma	Mid‐low	Yes, loop ileostomy	TEM	12 months	Successful closure
D'Ambrosio et al. [5], 2012	13	Surgery (*n* = 12); Radiation (*n* = 1)	Mid‐low	Yes, (*n* = 13)	TEM	25 months	93% Successful closure
Chen et al. [9], 2016	1	Trauma	Mid‐low	Yes, trasversostomy	TEM	12 months	Successful closure
van Vledder et al. [13], 2016	5	Surgery (*n* = 5)	Mid‐low	Yes (*n* = 3)	TEM or TAMIS	5 (1–68) months	40% Successful closure
Dapri et al. [10], 2017	1	Surgery	Mid‐low	Yes, loop ileostomy	TAMIS	2 months	Successful closure
Yuan et al., 2019	17	Surgery (*n* = 11); Congenital (*n* = 3); Obstetric (*n* = 2); IBDs (*n* = 1)	Mid‐low	Yes (*n* = 9)	TEO®	8 (2–24) months	82.4% Successful closure
Coratti et al. [11] 2019	1	Surgery	Mid‐low	Yes, loop ileostomy	TEO®	6 months	Successful closure
De Freitas et al. [12], 2019	1	Not reported	Mid‐low	Yes, loop ileostomy	R‐TAMIS	6 weeks	Successful closure
Wasilewski et al. [7], 2020	1	Surgery with radiation	Mid‐low	Yes, loop ileostomy	TAMIS	Not reported	—
Pellino et al. [8], 2020	1	Surgery	Mid‐low	Yes, diverting colostomy	R‐TAMIS	Not reported	—

Abbreviations: *R‐TAMIS*, robotic TAMIS; TAMIS, transanal endoscopic minimally invasive surgery; TEM, transanal endoscopic microsurgery; TEO®, transanal endoscopic operation.

According to the IDEAL framework, this technical note represents an IDEAL stage 1 innovation, aimed at demonstrating the technical feasibility and immediate safety of a fully transanal endoscopic approach for the repair of large, high post‐anastomotic rectovaginal fistulas. At this early stage, no claims regarding comparative effectiveness are intended.

## SURGICAL TECHNIQUE

A transanal endoscopic technique for high RVFs of large size is detailed in our technical video (Video 1).

**VIDEO 1:** Step‐by‐step demonstration of a fully transanal endoscopic approach for the closure of a large post‐anastomotic high rectovaginal fistula using the transanal endoscopic operation (TEO®) platform. The video illustrates patient positioning, defect marking, precise endoscopic dissection to achieve adequate reciprocal mobilization of the rectal and vaginal walls, and tension‐free layered closure of both defects. Video content can be viewed at https://onlinelibrary.wiley.com/doi/10.1111/codi.70419.

A 41‐year‐old obese woman with a complex surgical history was referred to our centre for restoration of bowel continuity after Hartmann procedure. Her initial oncological management had been performed in another institution, where she underwent endoscopic resection of a rectal well‐differentiated neuroendocrine tumour located 12 cm from the anal verge, with positive deep margins (R1). Histopathological analysis showed a grade 1 tumour (Ki‐67 index 1%, 2 mitoses/10 HPF), measuring 1.8 × 0.6 × 1.5 cm, infiltrating the submucosa, without necrosis. A subsequent 68Ga‐DOTATOC PET/CT demonstrated pathological uptake in a presacral nodule suspicious for nodal disease, and after multidisciplinary discussion surgical treatment was recommended. Final pathology after rectal resection showed no residual primary lesion and two positive lymph nodes (2/12), consistent with a well‐differentiated neuroendocrine tumour G1 (pT1b pN1), with R0 resection margins. A watch‐and‐wait follow‐up strategy was adopted, with no adjuvant treatment and no history of pelvic chemoradiotherapy.

**FIGURE 1:**
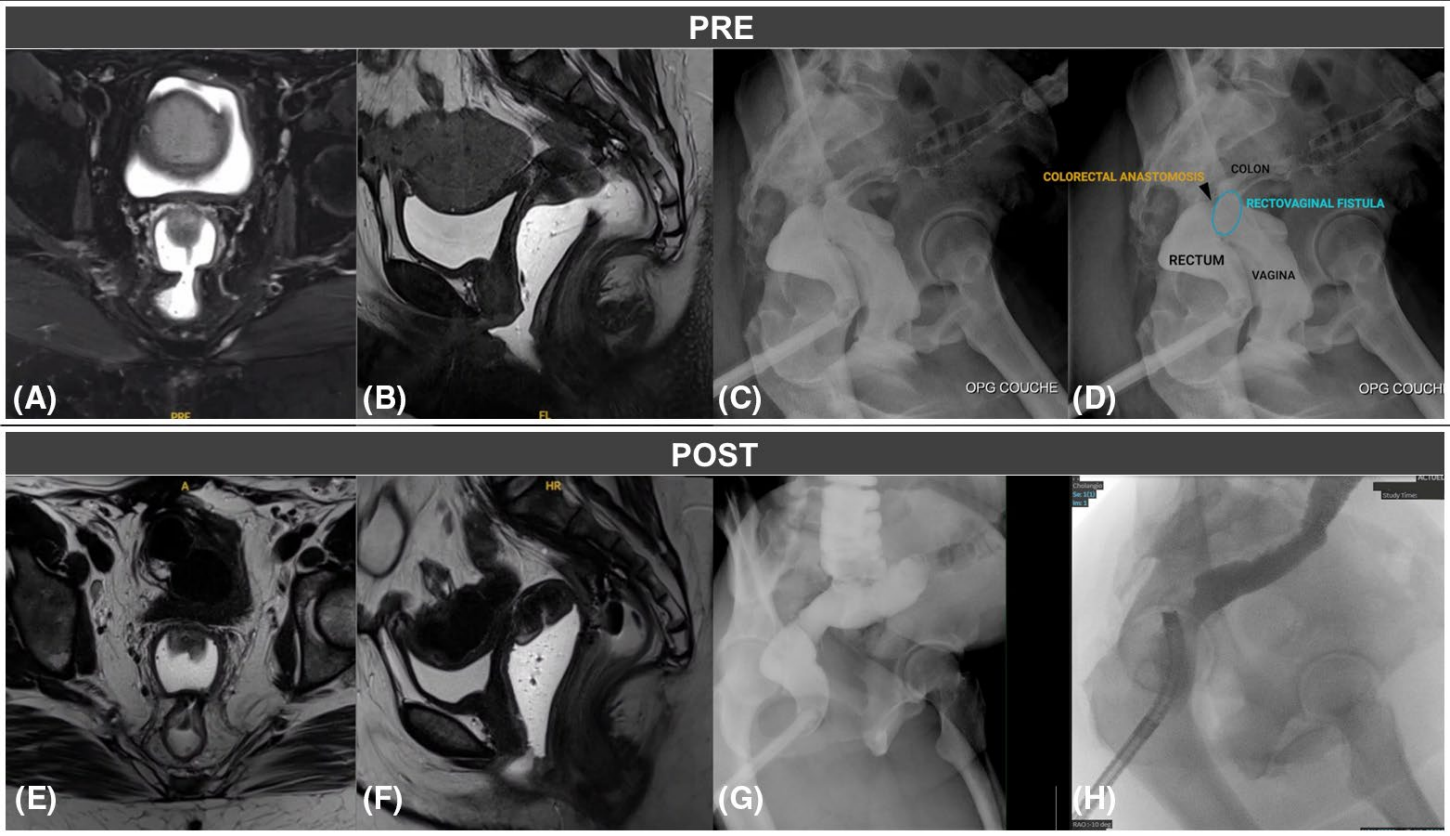
*PRE* preoperative pelvic magnetic resonance imaging (MRI)—axial (A) and sagittal (B) views—and lower GI series with rectal opacification (C and D) clearly showing a wide communication between the anterior aspect of the colorectal anastomosis and the posterior vaginal fornix; *POST* postoperative pelvic MRI—axial (E) and sagittal (F) views—and lower GI series with rectal opacification (G and H) clearly showing a successful closure of the rectovaginal communication.

The postoperative course of rectal resection was complicated by multiple abdominal reinterventions and ultimately a low Hartmann procedure. Due to a hostile abdomen and extensive pelvic adhesions, bowel continuity was restored in our centre with a colorectal anastomosis and protective ileostomy. A large rectovaginal fistula, measuring approximately 2 cm and located at 16–18 cm from the anal verge, subsequently developed at the level of the colorectal anastomosis. The diagnosis was confirmed by endoscopy, pelvic MRI and contrast studies (Figure 1A–D). Given the patient's complex surgical history, a fully transanal approach was proposed 3 years after the initial rectal surgery, with no evidence of local recurrence or distant disease at preoperative follow‐up. Written informed consent was obtained.

Six surgical steps are detailed as follows:

### Step 1: Patient position and endoscopic view

The patient was placed in a prone genu‐pectoral position under general anaesthesia. Two adhesive bands were put to separate the gluteus and expose the operative field. This position allows the target area to be positioned in the lower part (‘floor’) of the surgical field. This aspect is crucial as operating when the operative target is located at the top (‘ceiling’) of the surgical field is not only uncomfortable but also makes basic surgical actions like dissecting and suturing really challenging.

**FIGURE 2:**
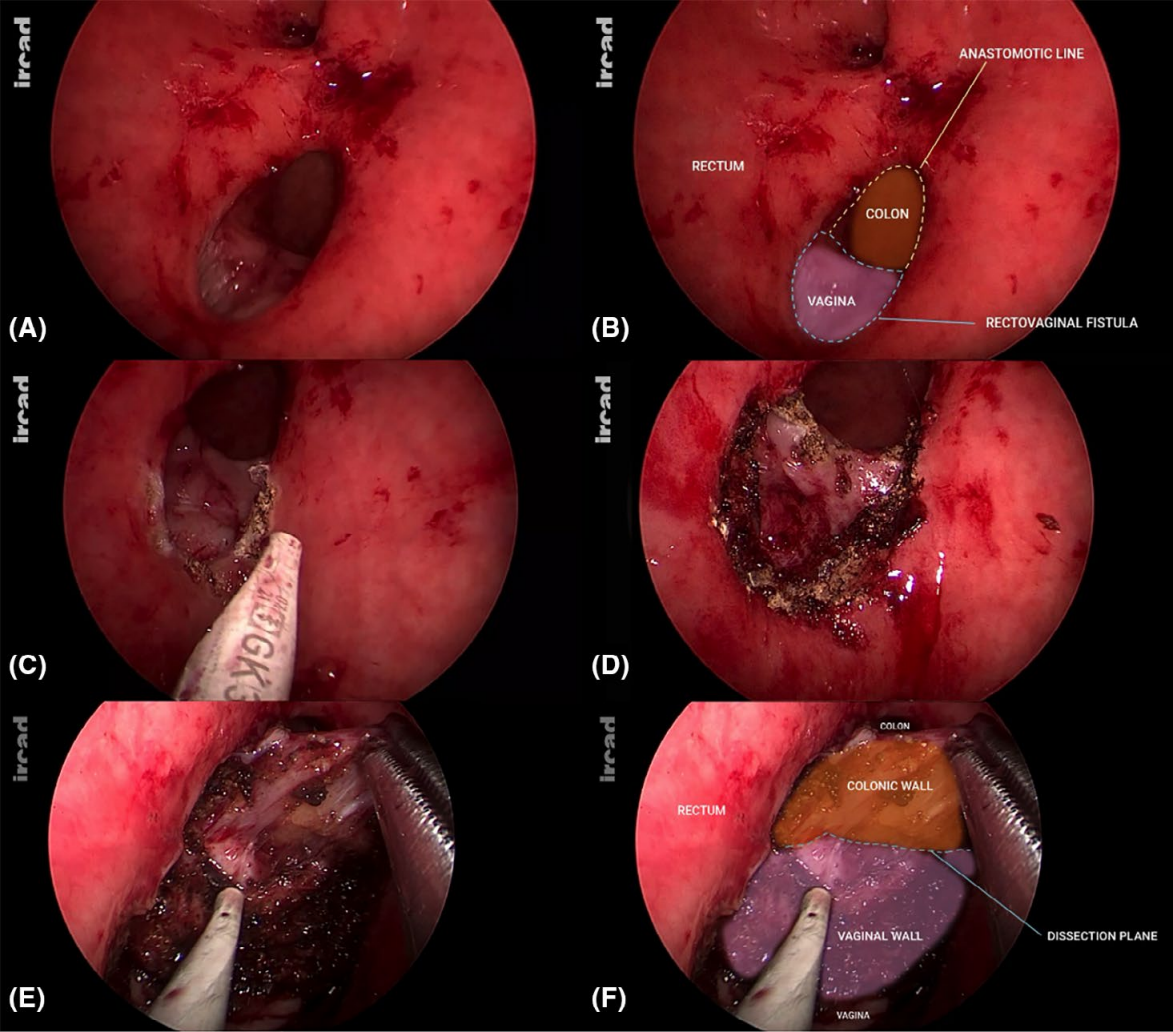
After introduction of the transanal endoscopic operation device and creation of a pneumorectum, endoscopic view showing the presence of a large rectovaginal fistula at the level of the anterior aspect of the colorectal anastomosis (A and B); the perimeter of the fistula's defect was marked with a monopolar Hook in order to guide the subsequent dissecting phase (C and D); dissection plane between vaginal wall and digestive structures was progressively developed all around the defect (E and F).

The TEO® device was introduced transanally after gradual anal dilatation while a sterile glove filled with gauzes was inserted in the vagina to prevent air leakage. After creation of a pneumorectum at 12 mmHg, endoscopic exploration confirmed the presence of a large rectovaginal fistula at the level of the colorectal anastomosis (Figure 2A,B).

### Step 2: Marking of the fistula orifice

The perimeter of the fistula's defect was marked with a monopolar Hook. This step must be performed before starting the dissection phase (Figure 2C,D). This manoeuvre allowed guiding the dissecting phase and avoiding getting lost throughout it.

### Step 3: Dissecting phase

Precise dissection was then performed all around the defect with the aim to separate, dissect and mobilize the vaginal stump walls from the colonic and rectal walls (Figure 2E,F). Several metallic anastomotic staples were identified on the vaginal side during dissection. Easily accessible staples were removed to facilitate the development of the rectovaginal space inferiorly and to obtain a good reciprocal mobilization of the two structures (rectum and vagina), respectively, while a few deeply embedded staples were left in place to avoid unnecessary tissue trauma.

Dissection was kept strictly within the rectovaginal plane under constant magnified endoscopic vision in order to avoid inadvertent entry into the peritoneal cavity and potential small‐bowel injury.

The rigid platform provides a stable magnified endoscopic view, facilitating precise and controlled dissection throughout this phase.

### Step 4: Assessment of proper reciprocal mobilization of vaginal and colonic and rectal walls

**FIGURE 3:**
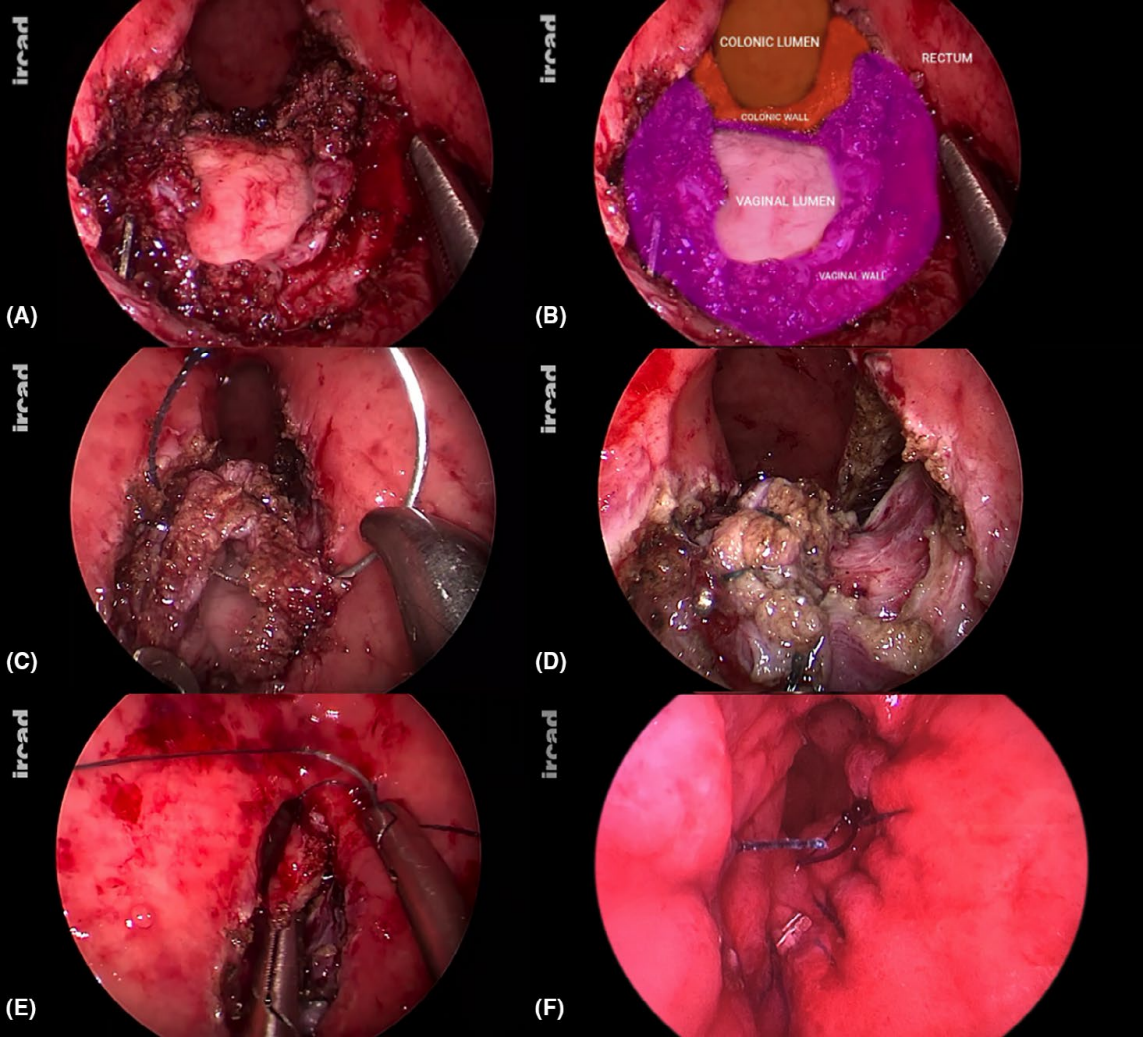
A good reciprocal mobilization is assessed ensuring at least 1 cm of release around the entire circumference of the defects before proceeding to suture phase (A, B); longitudinal closure of vaginal wall defect (C, D); anterior colorectal anastomotic aspect is restored by suturing in a transverse direction (E, F).

The goal of the dissection phase is to achieve reciprocal mobilization of the digestive and vaginal walls, ensuring at least 1 cm of release around the entire circumference of the defects (Figure 3A,B). Adequate mobilization of the vaginal walls was confirmed. This step is crucial to ensure tension‐free edges for suturing on both the vaginal (Figurand colorectal sides. Incomplete mobilization, especially in the case of large defects, may result in residual tension on the suture line, which could in turn predispose to fistula recurrence.

### Step 5: Vaginal wall closure

The vaginal defect was closed longitudinally with two barbed running sutures in a double layer. No tension was noted at the end of the sutures (Figure 3C,D). Metallic clips were used to secure the running suture. Transanal endoscopic suturing requires advanced hand–eye coordination within a narrow workspace, and adequate expertise is essential to ensure secure closure and procedural success.

### Step 6: Colorectal defect closure

The anterior aspect of the colorectal anastomosis was finally restored with two separated barbed running sutures. The transverse direction of the suture line and the use of interrupted running sutures were important to avoid the risk of stenosis (Figure 3E,F). Metallic clips were used to secure the running sutures.

Total operative time was 200 min, with negligible estimated blood loss. A urinary catheter was placed after induction of general anaesthesia and removed on postoperative day 1.

This was a 1‐day surgery procedure, and the postoperative course was uneventful. Postoperative imaging and endoscopic control at 3 months showed the successful closure of the defect with no residual communication (Figure 1E–H). Three months later, the ileostomy was subsequently reversed, and the patient made an uneventful recovery with no recurrence at 2 years after the procedure.

## DISCUSSION

RVFs represent one of the most challenging conditions in colorectal surgery because of their heterogeneity in aetiology, anatomy and degree of tissue damage. Surgical strategies vary according to type, size, location (mid‐low or high), and whether faecal diversion is used; in practice, the absence of standard classification and high recurrence rates emphasize the need for individualized management.

Mid‐low RVFs are often treated via transanal, transvaginal or transperineal techniques, including fistulectomy, advancement flap and Martius or muscle transposition, all carrying risks such as sphincter dysfunction or painful perineal incisions with prolonged recovery and local complications.

Notably, throughout the years, several minimally invasive and sphincter‐preserving techniques, including surgical or flexible endoscopic techniques, biomaterial closure and robotic transanal approaches, have emerged for mid‐low rectovaginal fistulas as valuable alternatives [14, 15].

In principle, the transanal route allows repairing the fistula on its high‐pressure side while a transvaginal route may be chosen in patients presenting rectal scarring (i.e. post radiotherapy, recurrent RVFs). In both cases, access may still be challenging when the fistula is high [3].

Flexible endoscopic solutions may theoretically reach high defects, but size often limits feasibility. Therefore, a transabdominal approach, often requiring faecal diversion, is normally chosen for higher and large RVFs, ranging from simple layered closure with omental interposition to partial or total rectal resection using a ‘pull‐through’ technique [1]. These approaches, however, are associated with considerable technical complexity and could be rather aggressive, especially in pluri‐operated patients, increasing the risk of collateral complications.

Despite numerous techniques, no universally accepted strategy exists, and surgical decisions rely on surgeon judgement based on anatomical and clinical variability.

In patients with hostile abdomens and multiple prior pelvic surgeries, transabdominal access may be associated with substantial morbidity, making a natural‐orifice transanal approach particularly appealing.

Originally conceived for local excision of early rectal neoplasms, the Transanal Endoscopic Microsurgery (TEM), the TEO® or the Transanal Endoscopic Minimally Invasive Surgery (TAMIS) platforms have evolved into versatile tools for complex pelvic reconstruction.

The use of transanal endoscopic platforms for RVF repair was first introduced by Darwood and Borley (2008), who described the successful treatment of a post‐anterior‐resection RVF using the TEMS platform, emphasizing the benefits of enhanced visualization and precise dissection in a minimally invasive setting, achieving rapid recovery and low morbidity [3].

Building on this, Vávra et al. reported a novel adaptation of TEM for rectovaginal fistulas, further validating its feasibility [2].

Several reports have since shown the efficacy of transanal endoscopic techniques for low–mid RVFs [5, 6, 7, 9, 10, 11, 13] (Table 1). D'Ambrosio et al. reported >92% success in 13 patients treated with TEM, emphasizing excellent exposure, minimal trauma and preserved anorectal function, although some steps required finger‐assisted dissection [5]. Chen et al. further demonstrated that TEM could be successfully used in recurrent cases after multiple failed procedures, highlighting the role of layered suturing and careful excision of scarred tissue [9]. Around the same period, van Vledder et al. reported their experience confirming the safety and efficacy of transanal endoscopic surgery (TES) using either the TEM or TAMIS platforms for the management of post‐anastomotic strictures, rectovaginal, and rectourinary fistulas. In their series, however, recurrence occurred in three of the five patients treated for RVFs, all of whom subsequently required more invasive transabdominal procedures. Despite these limitations, the authors concluded that transanal endoscopic repair should still be considered a first‐line option for localized postoperative complications [13].

More recently, Yuan et al. further expanded the minimally invasive paradigm, reporting favourable short‐term results with TEO®‐assisted rectal mucosal advancement flap (RMAF) for mid‐low RVFs [6]. In their cohort of 17 patients, short‐term outcomes were encouraging, although early recurrence occurred in 17.6% of cases (3/17), all of which presented chronic inflammation and tissue oedema around the fistula tract at index operation [6].

While most published experiences have focused on low‐ or mid‐level fistulas, our technique demonstrates that a high post‐anastomotic RVF, even of large size, can also be repaired transanally by a minimally invasive approach when appropriate expertise and endoscopic platforms are available.

Adequate mobilization of both rectal and vaginal walls under stable endoscopic vision assured by the TEO® platform enables a tension‐free, two‐layer closure, which is critical for durable healing [5, 13].

In their experience, Mukwege et al. described a laparoscopic minimally invasive strategy for high traumatic RVFs reporting a 90% success [1], confirming that even complex high‐level defects can be treated effectively using transabdominal minimally invasive access if precise dissection and layered closure are achieved. The present case extends this principle to a fully transanal route, supported by advanced endoscopic visualization and stable pneumorectum, allowing safe repair without the morbidity of transabdominal entry in a hostile abdomen.

In their recent work, Guadalajara et al. clearly illustrate how robotic platforms (rTAMIS) could further enhance transanal ergonomics and suturing dexterity [14]. Although not yet widely applied to RVF repair (only a few case reports in the literature [8, 12]), partly due to limited access to the technology, these systems could overcome some of the technical limitations of rigid platforms, particularly for high lesions or anatomically restricted pelvises.

In line with the most recent systematic review by Zeng et al., which analysed 71 patients treated with various endoscopic modalities, minimally invasive endoscopic repair achieved success rates ranging from 40% to 93%, with low complication rates and acceptable functional outcomes [15]. These data, combined with increasing experience from transanal platforms, support the growing role of endoscopic and robotic approaches in the tailored management of RVFs.

Given the rarity of high postoperative RVFs, evidence remains limited to case reports and small series. Nonetheless, our experience suggests that TEO®‐assisted transanal endoscopic repair may represent an organ‐preserving option even for selected large and high defects, particularly in previously operated abdomens. However, the transanal route remains technically demanding and should be reserved for carefully selected patients in experienced centres. Preoperative pelvic MRI and contrast studies are essential to define fistula anatomy and exclude complex tracts or suspected small bowel involvement. Intraoperatively, safe dissection relies on constant identification of the rectovaginal plane under stable magnified vision, avoiding blind traction or excessive energy use. In the presence of surrounding inflammation or suspected adhesions, maintaining continuous identification of the rectovaginal plane is crucial to prevent peritoneal breach and subsequent small‐bowel injury. If a clear dissection plane cannot be maintained or dense inflammatory adhesions are encountered, early conversion to a transabdominal approach should be considered to reduce the risk of collateral injury. In cases of small fistula orifices, limited enlargement of the mucosal opening may be required to allow adequate mobilization and tension‐free closure, but should be performed conservatively to preserve tissue vascularity. Finally, pelvic radiotherapy represents an additional challenge due to fibrosis and impaired healing; therefore, in irradiated patients this approach should be considered with caution and adjunctive strategies such as faecal diversion and/or tissue interposition may be required.

In our opinion, ideal candidates include patients with a single well‐defined high post‐anastomotic fistula, without active pelvic sepsis or previous pelvic radiotherapy, and in whom adequate mobilization can reasonably be expected.

Accordingly, given the complexity of the indication, these findings primarily demonstrate technical feasibility and safety and should not be interpreted as evidence of comparative effectiveness. Further evaluation in larger series is required to better define indications, reproducibility and long‐term outcomes.

## CONCLUSION

We describe an alternative approach to transabdominal surgery for treating high RVFs of large size using the TEO® platform. This strategy should be considered in patients with complex abdominal surgical histories who may benefit from a natural‐orifice, organ‐preserving solution without additional bowel resections. Transanal endoscopic RVF repair may be considered even for selected high and large defects, avoiding major rectal resection, but it requires advanced transanal dissection and suturing skills and should be performed in specialized centres.

## AUTHOR CONTRIBUTIONS

Conceptualization: Alfonso Lapergola, Antonio D'Urso, Didier Mutter. Methodology: Alfonso Lapergola, Federica Alicata, Antonio D'Urso, Didier Mutter. Data curation: Alfonso Lapergola, Federica Alicata, Paul Hag, Fatema Alhammadi, Pietro Riva, Elisa Reitano. Investigation: Alfonso Lapergola (performed the surgical procedure), Pietro Riva, Elisa Reitano. Validation: Alfonso Lapergola, Antonio D'Urso, Didier Mutter. Formal analysis: Alfonso Lapergola. Supervision: Alfonso Lapergola, Antonio D'Urso, Didier Mutter. Project administration: Alfonso Lapergola. Writing—original draft: Alfonso Lapergola, Federica Alicata, Paul Hag, Fatema Alhammadi. Writing—review and editing: Alfonso Lapergola, Pietro Riva, Elisa Reitano, Antonio D'Urso, Didier Mutter.

## FUNDING INFORMATION

This research did not receive any specific grant from funding agencies in the public, commercial or not‐for‐profit sectors.

## CONFLICT OF INTEREST STATEMENT

Authors have no financial/commercial interest to disclose.

## ETHICS STATEMENT

Ethical committee consent was not needed.

## PATIENT CONSENT

Patient written consent was obtained.

## Data Availability

The data that support the findings of this study are available from the corresponding author upon reasonable request.
